# SEVEN YEARS OF IMPLEMENTING AN INTERGENERATIONAL PROGRAM WITH UNIVERSITY/COMMUNITY PARTNERSHIPS

**DOI:** 10.1093/geroni/igac059.1056

**Published:** 2022-12-20

**Authors:** Skye Leedahl, Erica Estus, Kristin Fratoni Souza, Alexandria Capolino

**Affiliations:** University of Rhode Island, Kingston, Rhode Island, United States; University of Rhode Island, Kingston, Rhode Island, United States; University of Rhode Island, Kingston, Rhode Island, United States; University of Rhode Island, Kingston, Rhode Island, United States

## Abstract

The University of Rhode Island Engaging Generations Cyber-Seniors Program was first implemented in the fall semester of 2015. URI students from 15+ majors have supported the digital competency of older adults in Rhode Island each semester and summer session since its inception. Gradually over time, we modified and expanded this in-person program to meet student, older adult, community partner, and faculty needs. The pandemic led to our program expanding exponentially due to new grant funding opportunities, new partnerships, and student interest. This presentation will address strengths and challenges of implementing this program state-wide in a small, mostly urban state with community partners, mostly involving senior centers. We will describe moving from in-person to phone/virtual and now to a hybrid model. Last, we will explain the program’s efforts to conduct formative and summative evaluation research to assess program output and examine outcomes for students and older adults in the program.

